# Montreal Veterinary Medical Association

**Published:** 1892-01

**Authors:** A. S. Robertson

**Affiliations:** Secretary


*Montreal Veterinary Medical Association.*—The Montreal Veterinary Medical Association held its first meeting of the session 1891-92 on October 15th. The following were elected office-bearers for the ensuing year :

Dr. Charles McEachran, President, (re-elected) ; Dr. M. C. Baker, First Vice-President ; Dr. Mills, Second Vice-President ; Mr. A. T. Robertson, Secretary and Treasurer ; Mr. J. H. Seale, Librarian. Twenty-two new members were elected.

The second meeting of the Veterinary Medical Association for this session was held on October 29th, Dr. Charles McEachran occupying the chair. A vote of thanks was tendered Dr. Mills for his gift to the library of one of his recent works entitled "How to keep a dog in the city." A committee on experiments in drugs was formed. This committee to report the results of the experiments at the meetings of the Association, Mr. Ramsay reported a case of "Paralysis of Pelvic flexure of Large Colon."

The animal showed symptoms of indigestion or impaction. It responded quickly and freely to cathartics but four days afterwards showed every symptom of rupture.

Death : Post Mortem. Large accumulation in the pelvic flexure of great colon and a rupture of all its coats, which rupture on close examination proved to be ante mortem. At the inferior side of this large accumulation was a small opening about two inches in diameter which served for the intestinal contents to pass through without the mass being affected. The discussion of this case was heartily entered into by a large number of members, many of whom had seen similar cases. Dr. Baker related a case in which the symptoms much resembled those exhibited in Mr. Ramsay's case, although on making a post mortem examination he found the cæcum to be impacted.

Mr. Plaskett read a paper on "Glanders and Farcy." He took objection to Dr. Williams' statement that this disease might arise from surrounding septic conditions or as a complication to debilitating diseases, maintaining that these circumstances are merely predisposing causes decreasing the resistance of the animal to the attack of the germ. In speaking of the treatment of this disease he criticized the reported cures from intratracheal injection of iodine. The difficulty of diagnosis was fully discussed, the general opinion being that the production of the disease in other animals by inoculation was the only positive means of diagnosis.

The third meeting of the Association for this session was held on November 19. The President in the chair. Mr. Wells, Secretary of the committee on experiments in drugs reported having experimented with sulphate of eserine. The drug was tried on two different animals in different ways.

Animal No. 1. Normal condition of animal at 8.30 P. M. : pulse, 45 ; respiration, 12 ; temperature 100 4-5. One grain of eserine was dissolved and given in small venous injections every two minutes for a lapse of ten minutes at the end of which time the animal seemed to show uneasiness. Pulse not increased in frequency but stronger and fuller. Muscle tremors in shoulder and flank. Dilatation of pupil and very loud intestinal murmers. Five minutes after discontinuing the drug the pulse was 46, respiration 14, and temperature normal.

Animal No. 2. Not so strong as first one. Normal condition. Pulse, 42 ; temperature, 100 ; respiration, 8. At 9.10 P. M., gave one grain of sulphate of eserine in one injection in jugular vein. In fifteen minutes very pronounced muscular twitchings and tremors all over the body with excessive salivation, pupils dilated and constant champing of jaws.

At 9.30 P. M. there was a free evacuation of fæces with two more evacuations following at intervals of ten minutes. The committee also reported having made some experiments with chlorform as an anesthetic in the horse, but deferred their report till later as further investigations are under way when they hope to present some valuable deductions as to chloroform and ether anesthesia.

Mr. Seale reported a case of ''rupture of the stomach '' which presented all the usual symptoms and was diagnosed as such. The post mortem showed the duodenum and pyloric end of stomach to be completely obstructed by bots. The rupture was located in the greater curvature towards the cardiac end. There was an animated discussion in which it was shown that vomition in the horse was possible without the presence of rupture. Dr. Charles McEachran reported a case of a horse belonging to the Shedden Co. that was subject to occasional attacks of colic every attack being attended with vomition.

A paper on Laminitis was read by Mr. Ramsay who took the view that laminitis was more a secondary than a primary affection, being preceeded by general indisposition or some internal febrile condition. A lively discussion on treatment ensued. The essayist's recommendation of hot fomentations in the congestive stage was criticized, several of the members claiming that cold would have a similar effect.

A. S. Robertson, *Secretary*,

